# Morbidity and mortality reduction associated with polysomnography testing in idiopathic pulmonary fibrosis: a population-based cohort study

**DOI:** 10.1186/s12890-021-01555-x

**Published:** 2021-06-02

**Authors:** Nicholas T. Vozoris, Andrew S. Wilton, Peter C. Austin, Tetyana Kendzerska, Clodagh M. Ryan, Andrea S. Gershon

**Affiliations:** 1 Division of Respirology, Department of Medicine, St. Michael’s Hospital, 30 Bond Street, Toronto, ON M5B 1W8 Canada; 2 Keenan Research Centre in the Li Ka Shing Knowledge Institute, St Michael’s Hospital, Toronto, ON Canada; 3 Department of Medicine, University of Toronto, Toronto, ON Canada; 4 ICES (Formerly Known As Institute for Clinical Evaluative Sciences), Toronto, ON Canada; 5 Institute of Health Policy, Management, and Evaluation, University of Toronto, Toronto, ON Canada; 6 Department of Medicine, The Ottawa Hospital Research Institute, University of Ottawa, Ottawa, ON Canada; 7 Division of Respirology, University Health Network, Toronto, ON Canada; 8 Division of Respirology, Sunnybrook Health Sciences Centre, Toronto, ON Canada

**Keywords:** Sleep testing, Sleep breathing disorder, Pulmonary fibrosis, Health outcomes research, Health administrative database research

## Abstract

**Background:**

It is not well-known if diagnosing and treating sleep breathing disorders among individuals with idiopathic pulmonary fibrosis (IPF) improves health outcomes. We evaluated the association between receipt of laboratory-based polysomnography (which is the first step in the diagnosis and treatment of sleep breathing disorders in Ontario, Canada) and respiratory-related hospitalization and all-cause mortality among individuals with IPF.

**Methods:**

We used a retrospective, population-based, cohort study design, analyzing health administrative data from Ontario, Canada, from 2007 to 2019. Individuals with IPF were identified using an algorithm based on health administrative codes previously developed by IPF experts. Propensity score matching was used to account for potential differences in 41 relevant covariates between individuals that underwent polysomnography (exposed) and individuals that did not undergo polysomnography (controls), in order minimize potential confounding. Respiratory-related hospitalization and all-cause mortality were evaluated up to 12 months after the index date.

**Results:**

Out of 5044 individuals with IPF identified, 201 (4.0%) received polysomnography, and 189 (94.0%) were matched to an equal number of controls. Compared to controls, exposed individuals had significantly reduced rates of respiratory-related hospitalization (hazard ratio [HR] 0.43, 95% confidence interval [CI] 0.24–0.75), p = 0.003) and all-cause mortality (HR 0.49, 95% CI 0.30–0.80), p = 0.004). Significantly reduced rate of respiratory-related hospitalization (but not all-cause mortality) was also observed among those with >  = 1 respiratory-related hospitalization (HR 0.38, 95% CI 0.15–0.99) and systemic corticosteroid receipt (HR 0.37, 95% CI 0.19–0.94) in the year prior to the index date, which reflect sicker subgroups of persons.

**Conclusions:**

Undergoing polysomnography was associated with significantly improved clinically-important health outcomes among individuals with IPF, highlighting the potential importance of incorporating this testing in IPF disease management.

**Supplementary Information:**

The online version contains supplementary material available at 10.1186/s12890-021-01555-x.

## Introduction

Idiopathic pulmonary fibrosis (IPF) is the most common fibrotic lung disease and its prevalence may be increasing [1, 2]. IPF is generally a progressive disease, with a median survival from diagnosis of 2–3 years [3, 4]. Acute respiratory deteriorations occur in IPF, due to a known cause (like respiratory tract infection) or due to an unknown cause (termed ‘acute exacerbations’) [3, 4]. Acute exacerbations are the leading cause of hospitalization in IPF (5,6). Recently introduced anti-fibrotic drug therapies for IPF are successful in slowing disease progression, but are not curative [7, 8]. Although lung transplantation is a definite treatment for IPF, availability is limited to a small percentage of individuals due to limited organ supply [9].

Sleep breathing disorders, including obstructive sleep apnea (OSA) [10–16] and sustained nocturnal hypoxemia [11, 17], are commonly encountered in IPF. In advanced IPF, hypoventilation may also occur, but the prevalence of this sleep breathing disorder in IPF is not well-known. Untreated sleep breathing disorders may worsen IPF through several mechanisms. First, chronic, intermittent hypoxemia has been shown in animal models to promote pulmonary fibrosis through oxidative and inflammatory pathways [18, 19]. Second, repetitive forced inspirations against a closed glottis, which occurs in OSA, may cause recurrent tractional injury to peripheral lung tissue, which may in turn promote pulmonary fibrosis [20]. Third, gastroesophageal reflux, which can be induced by OSA [21], may lead to the development or progression of IPF [22, 23]. Finally, untreated OSA may contribute to complications of pulmonary arterial hypertension [24], the presence of which is associated with increased mortality in IPF [25]. Although sleep breathing disorders may theoretically worsen IPF, there is a paucity of published literature on whether diagnosing and treating sleep breathing disorders in the setting of IPF influences health outcomes. Two small, observational studies involving individuals with newly-diagnosed IPF and moderate-to-severe OSA showed that those adherent with positive airway pressure (PAP) therapy had significantly better survival than those that were non-adherent [14, 15], but that there was no improvement in exacerbations necessitating hospitalization [14]. A final observational study involving individuals with a variety of forms of interstitial lung disease (only 32.5% of whom had IPF) found no improvement in all-cause mortality or progression-free survival among those with OSA versus no OSA, nor among those with OSA adherent with PAP compared to those with OSA and not using PAP [26]. However, in the subset of individuals with interstitial lung disease requiring supplemental oxygen, adherence to PAP therapy for OSA was associated with significantly better progression-free survival [26]. The need for further research to clarify the importance of diagnosing and treating sleep breathing disorders in IPF has been advocated by international IPF guidelines [3].

In Ontario, Canada, sleep breathing disorders are diagnosed solely via laboratory-based polysomnography (PSG), with testing mandated prior to the initiation of appropriate treatments. Therefore, we considered PSG receipt a surrogate marker for the diagnosis and treatment of sleep breathing disorder for the purposes of this study. The objective of our study was to evaluate the association between receipt of PSG and respiratory-related hospitalization and mortality among individuals with IPF. Our hypothesis was that individuals with IPF that undergo PSG (a proxy marker for the diagnosis and treatment of sleep breathing disorder) will have reduced respiratory-related hospitalization and mortality than those that do not undergo such testing. Our work was intended to be hypothesis-generating.

## Methods

### Study design

This was a retrospective cohort study. We analyzed health administrative data housed at ICES (formerly known as Institute for Clinical Evaluative Sciences) for the province of Ontario, Canada (13.5 million people), for the period April 1, 2007 to March 31, 2019. Because all residents of Ontario have public health insurance, with a single payer for all medically necessary health services, our analyses are population-based. ICES is a prescribed entity under Section 45 of Ontario’s Personal Health Information Protection Act. Section 45 authorizes ICES to collect personal health information for the purpose of analysis or compiling statistical information with respect to the management of, evaluation or monitoring of, the allocation of resources to or planning for all or part of the health system. This project was conducted under Section 45 and received approval from ICES’ Privacy and Legal Office. This project was also approved by the Research Ethics Board at Sunnybrook Health Sciences Centre, Toronto, Canada.

### Data sources

Using unique encoded identifiers, multiple Ontario health care administrative databases were linked and analyzed at ICES, including: the Canadian Institute for Health Information Discharge Abstract database (CIHI-DAD) (contains information on all hospital discharges); the National Ambulatory Care Reporting System (NACRS) database (contains information on emergency room (ER) and hospital-based clinic visits); the Ontario Health Insurance Plan (OHIP) claims database (contains information on all physician fee-for-service patient care claims, in both ambulatory and hospital settings); the Ontario Drug Benefit (ODB) database (contains information on all publicly-funded, outpatient drug dispensings to individuals aged 65 years and older); and, the Registered Persons Database (contains information on demographics and mortality). Other databases that were used are outlined in the Additional file 1.

### Study population

Ontario residents with a diagnosis of IPF aged 66 years and older between April 1, 2007 and December 31, 2017 were considered. We identified individuals with IPF from health administrative data, using an algorithm developed by a group of internationally-recognized IPF experts [1] that, while non-validated, has been previously applied in multiple published studies [1, 27–29]. According this algorithm [1, 27–29], individuals were considered to have IPF if the following three criteria were met: 1) there was at least one International Classification of Diseases Version 10 (ICD-10) coding for J84.1 (codes for IPF and usual interstitial pneumonia) in either Canadian Institute for Health Information Discharge Abstract Database (CIHI-DAD) or National Ambulatory Care Reporting System (NACRS) between April 1, 2007 and December 31, 2017; and, 2) there was at least one claim for either a computed tomography chest scan, or a lung biopsy (including transbronchial biopsy, surgical lung biopsy, or endobronchial ultrasound and biopsy), or a bronchoscopy, in the Ontario Health Insurance Plan (OHIP) (see Additional file 1 for relevant codes), prior to the last J84.1 coding (with a maximum look-back to April 1, 2006); and, 3) there was no coding for other forms of interstitial lung disease (see Additional file 1 for relevant codes) in either CIHI-DAD or NACRS within the 12 months after the last J84.1 coding (with a maximum follow-up date of December 31, 2018). Although individuals with IPF younger than 66 years old were excluded from this study (because drug dispensing data were not available for them in the Ontario Drug Benefit database and we considered it important to adjust our analyses for receipt for pharmacotherapies), IPF is a disease of older adults, with an estimated 70% or more of affected individuals being older than age 65 years [2, 29].

Two exclusion criteria were applied. First, individuals receiving palliative care (based on physician service and hospitalization codes) in the year prior to the index date (defined below) were excluded, as individuals receiving such care are more likely to have poor health outcomes and less likely to undergo PSG, and their inclusion could serve to potentially introduce bias. Second, individuals that in the five years prior to the index date (defined below) underwent any PSG, or received PAP therapy, or received home supplemental oxygen, were excluded. These groups were excluded because they may have already acquired health benefits from having sleep breathing disorder diagnosed and treated, and if not excluded, their presence could then potentially introduce bias.

### Group and index date definitions

#### Exposed group

An individual was classified as exposed if the following two criteria were met: 1) there was an OHIP claim for any PSG (see Additional file 1 for relevant codes) between April 1, 2007 and December 31, 2017, after the first J84.1 coding; and, 2) there was an OHIP claim for spirometry (see Additional file 1 for relevant codes) within the 12 months preceding the PSG. The latter criterion was included in order to ensure that both exposed and control individuals underwent spirometry, as controls were identified by receipt of this testing (further details outlined below) and since undergoing spirometry may influence health outcomes in IPF. Only laboratory-based (and not home-based) PSG designated exposed group classification for the following reasons: home-based testing is not currently reimbursed by OHIP, and therefore, few, if any, individuals were anticipated to have received it; in Ontario, it is mandated that prescription of any home PAP therapy be supported by a laboratory-based PSG (30); and, clinical practice guidelines do not recommend home-based sleep testing for individuals with chronic respiratory disease (like IPF) [31, 32]. If an individual underwent more than one PSG within the study accrual period, then only the first one was considered. The index date was 3 months after the date of the first PSG. The rationale for the index date being set 3 months after the PSG date, and not sooner, was to allow individuals a reasonable amount of time following their PSG to see a physician regarding the results and have possible treatment initiated.

Although receipt of certain forms of sleep breathing disorder therapy (i.e., PAP and supplemental oxygen) is partially recorded in Ontario health administrative databases, this was intentionally not selected as the exposure for several reasons. First, OSA may be reasonably treated in some individuals with either weight reduction, positional therapy or a mandibular advancement device, and receipt of these therapies are not captured in our health administrative databases. Individuals receiving such therapies would be erroneously classified as controls, if receipt of PAP and/or supplemental were selected as the exposure. Second, because there is incomplete recording of PAP therapy receipt in our health administrative databases, the control group could be contaminated by exposed individuals, had PAP receipt been selected as the exposure.

#### Control group

Individuals in the control group did not undergo any PSG between April 1, 2007 and December 31, 2017. Individuals entered the control group by receiving spirometry at least once between April 1, 2007–December 31, 2017 after the first J84.1 coding. Receipt of an investigation was intentionally selected to define control group entry in order to minimize bias, since exposed group entry involved investigation receipt (i.e., PSG). Spirometry was selected as the testing for control group designation, since this is commonly performed test in IPF for both diagnostic and follow-up reasons. If spirometry had been received more than once by controls during the accrual period, a spirometry receipt date was randomly selected for such individuals, and then based on the time distribution of spirometry receipt to PSG receipt in the exposed group, a random date following that distribution was assigned to controls after the spirometry receipt date. Using this approach, a fictitious PSG date was in effect created for each control. The index date was 3 months after the fictitious PSG date, consistent with the approach used for the exposed group.

#### Outcomes

**Fig. 1:**
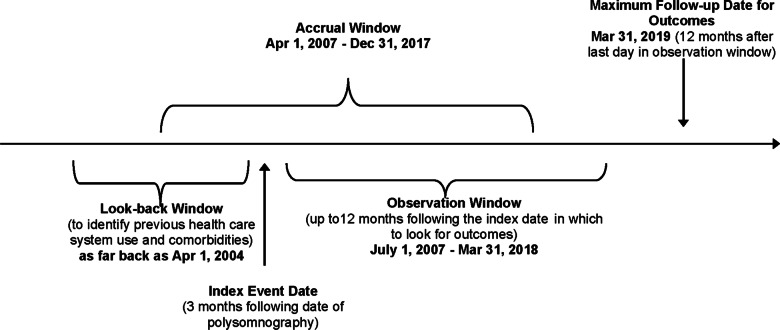
Study time frames

Respiratory-related hospitalization was the primary outcome, since this is a clinically-important event among individuals with IPF, associated with high mortality risk [5, 6]. All-cause mortality was a secondary outcome. Respiratory-related hospitalization was defined by one of the following ICD-10 codes being recorded in CIHI-DAD as the reason for hospitalization: J84.1 (interstitial pulmonary disease); J96 (respiratory failure); J09-18, J20-22 and J40 (pneumonia); and, I27.0, I27.2 and I27.9 (pulmonary hypertension). Pneumonia and pulmonary hypertension were included as reasons for respiratory-related hospitalization, since these respiratory pathologies are known to occur in IPF, may necessitate hospitalization, and are associated with increased mortality risk [5, 6, 25]. All outcomes were evaluated up to 12 months after the index date [with the latest possible follow-up date of March 31, 2019, assuming a PSG date of as late as December 31, 2017, which would then result in an index date of March 31, 2018 (Fig. 1 depicts study time frames)], or up to the date of death, or up to date of lung transplantation (see Additional file 1 for definition), whichever came first. Individuals were censored on the date of lung transplantation, because risk for IPF-related morbidity and mortality was anticipated to dramatically differ post-lung transplantation.

#### Propensity score matching

Propensity score matching was used to create matched samples of exposed and control individuals on baseline sociodemographic and health characteristics to reduce bias [33]. A 1:1 matching ratio was selected, since this was previously shown to minimize bias and inclusion of more controls results in minimal precision increase [34]. Following previously published recommendations, individuals were matched on the logit of the propensity score using a width caliper equal to 0.2 of the standard deviation of the logit of the propensity score [35]. A propensity score for PSG receipt was developed using logistic regression modelling incorporating 41 variables, including multiple markers of IPF severity (such as, respiratory-related hospitalization (defined above) in the year prior to the index date, intensive care unit (ICU) admission during a respiratory-related hospitalization in the year prior to the index date, physician-diagnosed congestive heart failure [CHF], and systemic corticosteroid or respiratory antibiotic receipt in the year prior to the index date), general health status, comorbidities, health care system utilization, relevant prescription medication receipt and demographics. A full list of variables included in the propensity score model can be found in the Additional file 1. Exposed and control individuals were matched at the index date on the propensity score, as well as on the following variables in order to facilitate planned sensitivity analyses (described below): respiratory-related hospitalization in the year prior to the index date; CHF diagnosis prior to the index date; systemic corticosteroid receipt in the year prior to the index date; and, sex.

**Table 1:** Cohort baseline characteristics, before and after propensity score matching (abridged version^*^)

Baseline characteristics	Prior to propensity score matching			After propensity score matching		
	Exposed N = 201	Controls N = 4843	Standardized difference^†^	Exposed N = 189	Controls N = 189	Standardized difference^†^
Age (mean + SD)	75.9 ± 6.2	78.3 ± 6.8	0.37	76.2 ± 6.1	76.4 ± 6.8	0.04
Women (%)	33.8	45.1	0.23	33.3	33.3	0.00
Respiratory-related hospitalization past year (%)	19.9	9.1	0.31	18.5	18.5	0.00
ICU admission during respiratory-related hospitalization past year (%)	4.0	1.5	0.15	4.2	4.8	0.03
Congestive heart failure (%)	46.3	29.8	0.34	45.5	45.5	0.00
Systemic corticosteroid receipt past year (%)	37.3	33.0	0.09	37.0	37.0	0.00
Respiratory antibiotic receipt past year (%)	72.1	66.5	0.12	72.0	73.5	0.04
Anti-fibrotic drug^§^ receipt past year (%)	^‡^	0.9	0.09	^‡^	^‡^	0.00
Total number outpatient visits past year (mean + SD)	19.2 ± 10.2	15.7 ± 9.2	0.36	18.9 ± 10.0	18.1 ± 9.3	0.09
CT Chest scan past year (%)	67.7	54.7	0.27	66.7	69.3	0.06
Echocardiogram past year (%)	65.7	43.8	0.45	64.0	64.6	0.01
Exercise oximetry past year (%)	38.3	31.8	0.14	37.6	37.6	0.00
Pulmonary embolism^ǁ^ (%)	6.0	3.2	0.13	6.3	6.9	0.02
COPD (%)	70.6	67.6	0.07	69.8	68.8	0.02
GERD^ǁ^ (%)	6.5	5.2	0.06	6.3	5.8	0.02
Myocardial infarction (%)	14.9	10.0	0.15	14.3	16.4	0.06
Other pulmonary disease^ǁ^^¶^ (%)	94.5	87.4	0.25	94.2	94.7	0.02
Opioid receipt past 3 months (%)	17.9	17.8	0.00	19.0	19.0	0.00
Diuretic medication receipt past 3 months (%)	36.8	23.8	0.29	34.4	37.0	0.06
Other cardiac drug^**^ receipt past 3 months (%)	77.6	67.3	0.23	77.2	77.8	0.01

COPD = chronic obstructive pulmonary disease; GERD = gastroesophageal reflux; ICU = intensive care unit; ODB = Ontario Drug Benefit; SD = standard deviation

^*^A full list of the variables included in the propensity score model can be found in the Additional file 1

^†^Standardized differences of > 0.10 are thought to indicate potentially meaningful differences

^‡^Data has been suppressed, according to ICES guidelines, because of small sample size

^§^Includes Pirfenidone and Nintedanib

^ǁ^Presence of comorbidities was based on 3-year look-back from the index date

^¶^Includes asthma, bronchiectasis, occupational lung disease, pleural effusion, interstitial disease, pneumothorax, atelectasis and other

^**^Includes beta-blockers, calcium channel blockers, angiotensin converting enzyme inhibitors (ACEI), and angiotensin receptor blockers (ARB)

#### Statistical analysis

To assess the adequacy of the matching process, standardized differences comparing the distribution of each of the covariates between the exposed and control groups were calculated before and after propensity score matching [36]. For the respiratory-related hospitalization outcome, hazard ratios (HR) with 95% confidence intervals (CI) were calculated using cause-specific modelling that accounted for the competing risk of death. For all-cause mortality, a Cox model was used to regress the hazard of death on exposure status. All regression models used a robust variance estimator [37]. Number needed to treat (NNT) was estimated by calculating the inverse of the absolute risk difference. Cumulative incidence function curves were estimated for respiratory-related hospitalization (where the competing risk death was adjusted for) and Kaplan–Meier curves were estimated for all-cause mortality.

#### Sensitivity analyses

History of respiratory exacerbation, CHF complication and systemic corticosteroid receipt are all considered markers of IPF severity [5, 6, 38]. Therefore, outcomes were examined stratifying by each of these variables separately, in order to further minimize confounding by indication by evaluating outcomes among healthier subsets of persons, and to further minimize 'healthy user' bias by evaluating outcomes among sicker subgroups of individuals. Additional sensitivity analyses are outlined in the Additional file 1. The propensity score was re-estimated for each specific sensitivity analysis.

## Results

### Derivation and description of the cohort

**Fig. 2:**
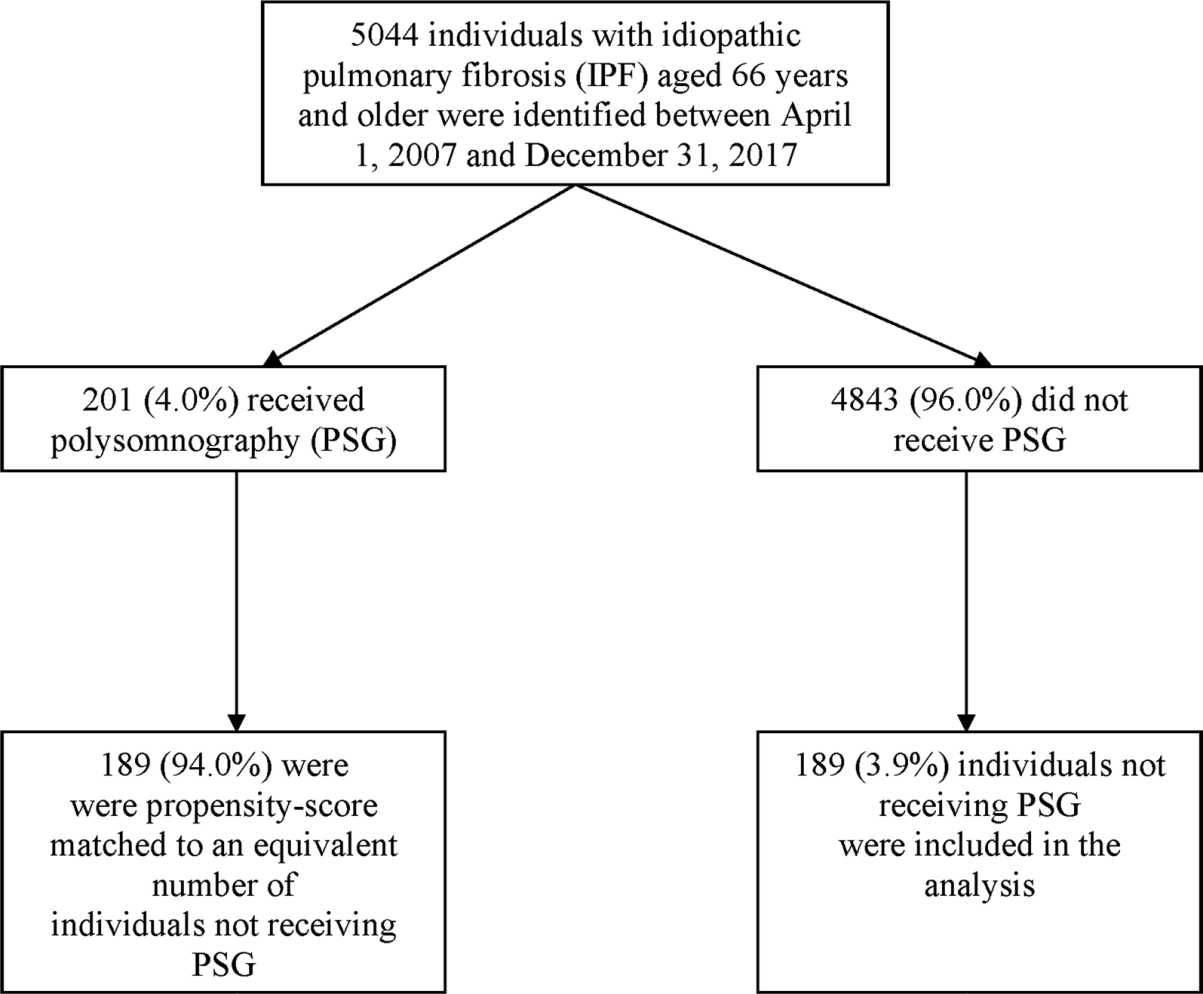
Flow diagram outlining exposed and control group identification

There were 5044 individuals with IPF identified, aged 66 years and older, of whom 201 (4.0%) received PSG during the accrual period (Fig. 2). Out of this group, 189 (94.0%) exposed individuals were matched to an equal number of controls. Before propensity score matching, compared to the control group, the exposed group had a younger mean age and consisted of a greater proportion of men and rural residents, had a smaller percentage of low income individuals, and multiple markers of IPF severity were more prevalent (such as, being admitted to hospital or ICU for respiratory-related reasons in the preceding year, having CHF, having other comorbidities, and systemic corticosteroid and respiratory antibiotic receipt). After propensity score matching, exposed and control individuals were adequately balanced on baseline characteristics, with standardized differences being below 10% for all variables, except five (rural residence, diabetes, kidney disease, antiplatelet/anticoagulant drug receipt, and year of cohort entry), where trivial imbalance remained (standardized differences ranged from 11 to 17%) (Table 1 and Additional file 1).

### Main analysis

**Fig. 3:**
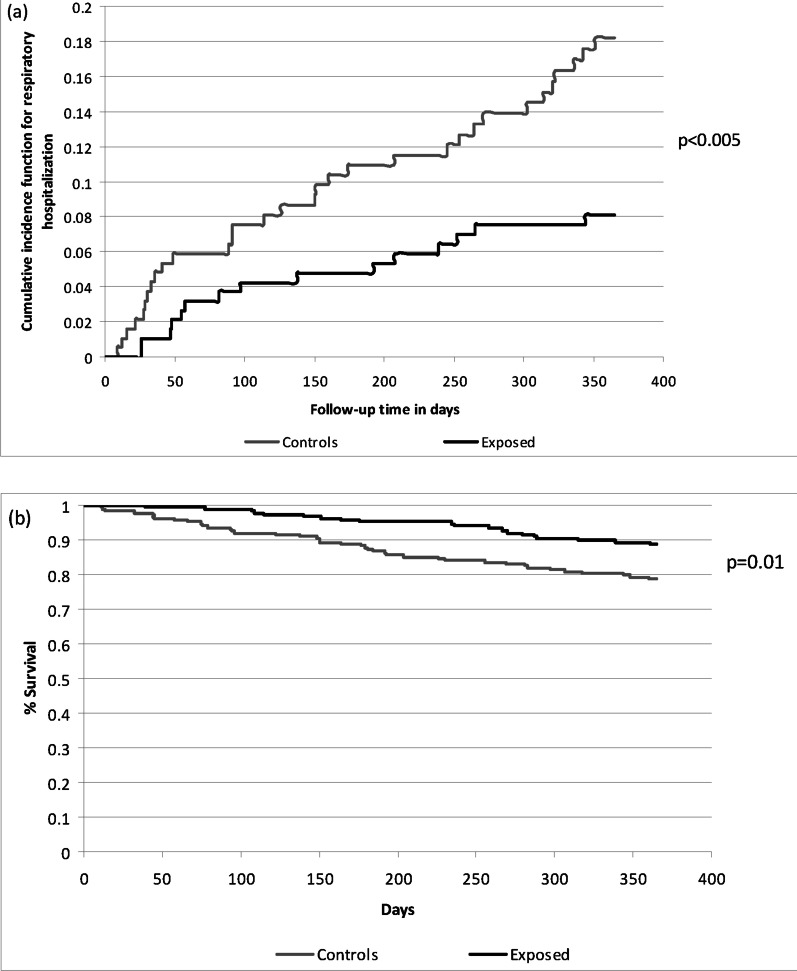
**a** Cumulative incidence function curves for respiratory-related hospitalization and **b** Kaplan–Meier curves for all-cause mortality

In the propensity score matched cohort, compared to controls, individuals undergoing PSG had significantly reduced rates of respiratory-related hospitalization (HR 0.43, 95% CI 0.24–0.75, p = 0.003, NNT 11) and all-cause mortality (HR 0.49, 95% CI 0.30–0.80, p = 0.004, NNT 10) (Table 2). The cumulative incidence of respiratory-related hospitalization was significantly lower and the probability of survival was significantly higher among individuals undergoing PSG versus controls (Fig. 3).

**Table 2:** Hazard ratios (HR) and confidence intervals (CI) for outcomes in propensity-score matched cohort (main analysis)

Outcome	Exposure status	Number of events (%)	HR (95% CI)	p-value
Respiratory-related hospitalization	Exposed	15 (7.9)	0.43 (0.24–0.75)	0.003
	Controls	32 (16.9)	1.00	
All-cause mortality	Exposed	21 (11.1)	0.49 (0.30–0.80)	0.004
	Controls	40 (21.2)	1.00

### Sensitivity analyses

#### By respiratory-related hospitalization

In the subgroup of individuals that experienced no respiratory-related hospitalization in the year prior to the index date, compared to controls, individuals undergoing PSG had significantly reduced rate of all-cause mortality (HR 0.34, 95% CI 0.23–0.83, p = 0.01), but not respiratory-related hospitalization (Table 3). In the subgroup of individuals with ≥ 1 respiratory-related hospitalization in the year prior to the index date, significantly reduced rate of respiratory-related hospitalization (HR 0.38, 95% CI 0.15–0.99, p = 0.05) was observed among individuals undergoing PSG relative to controls, but there was no significant difference all-cause mortality rate.

**Table 3:** Hazard ratios (HR) and confidence intervals (CI) for outcomes in propensity-score matched cohort, stratifying by respiratory-related hospitalization

Respiratory-related hospitalization status	Outcomes	Exposure status	Number of events (%)	HR (95% CI)	p-value
No respiratory-related hospitalization in year prior to index date	Respiratory-related hospitalization	Exposed	9 (5.9)	0.47 (0.21–1.06)	0.07
		Controls	18 (11.8)	1.00	
	All-cause mortality	Exposed	14 (9.2)	0.34 (0.23–0.83)	0.01
		Controls	30 (19.6)	1.00	
≥ 1 respiratory-related hospitalization in year prior to index date	Respiratory-related hospitalization	Exposed	^*^	0.38 (0.15–0.99)	0.05
		Controls	10 (34.5)	1.00	
	All-cause mortality	Exposed	^*^	0.42 (0.17–1.05)	0.06
		Controls	10 (34.5)	1.00	

^*^Data has been suppressed, according to ICES guidelines, because of small sample size

#### By CHF

Compared to controls, there were no significant differences in rates of respiratory-related hospitalization or all-cause mortality among those undergoing PSG, in both the subgroup without CHF complication and in the subgroup with CHF complication (Table 4).

**Table 4:** Hazard ratios (HR) and confidence intervals (CI) for outcomes in propensity-score matched cohort, stratifying by congestive heart failure (CHF)

Sex	Outcomes	Exposure status	Number of events (%)	HR (95% CI)	p-value
Without CHF	Respiratory-related hospitalization	Exposed	9 (8.7)	0.57 (0.25–1.33)	0.19
		Controls	15 (14.4)	1.00	
	All-cause mortality	Exposed	10 (9.6)	0.75 (0.33–1.72)	0.49
		Controls	13 (12.5)	1.00	
With CHF	Respiratory-related hospitalization	Exposed	6 (7.1)	0.41 (0.16–1.05)	0.06
		Controls	14 (16.7)	1.00	
	All-cause mortality	Exposed	9 (10.7)	0.45 (0.20–1.02)	0.06
		Controls	19 (22.6)	1.00	

#### By systemic corticosteroid receipt

In the subgroup of individuals with no systemic corticosteroid receipt in the year prior to the index date, there were no significant differences among individuals having undergone PSG versus controls in respiratory-related hospitalization and all-cause mortality rates (Table 5). In the subgroup of individuals that received a systemic corticosteroid in the year prior to the index date, there was significantly lower rate of respiratory-related hospitalization (HR 0.37, 95% CI 0.14–0.94, p = 0.04), but not all-cause mortality, among those undergoing PSG relative to controls.

**Table 5:** Hazard ratios (HR) and confidence intervals (CI) for outcomes in propensity-score matched cohort, stratifying by systemic corticosteroid receipt

Sex	Outcomes	Exposure status	Number of events (%)	HR (95% CI)	p-value
No corticosteroid receipt in year prior to index date	Respiratory-related hospitalization	Exposed	8 (6.8)	0.54 (0.23–1.30)	0.17
		Controls	14 (11.9)	1.00	
	All-cause mortality	Exposed	10 (8.5)	0.47 (0.22–1.02)	0.06
		Controls	20 (17.0)	1.00	
Corticosteroid receipt in year prior to index date	Respiratory-related hospitalization	Exposed	6 (8.6)	0.37 (0.14–0.94)	0.04
		Controls	15 (21.4)	1.00	
	All-cause mortality	Exposed	9 (12.9)	0.57 (0.25–1.28)	0.17
		Controls	15 (21.4)	1.00	

## Discussion

Our population-level study demonstrates the novel and important finding that undergoing PSG is associated with significantly lower rates of respiratory-related hospitalization and all-cause mortality among individuals with IPF. The credibility of our findings is further corroborated by significantly reduced respiratory-related hospitalization in association with PSG receipt amongst sicker subgroup of individuals, like those with prior history of respiratory-related hospitalization and prior systemic corticosteroid receipt. The finding that only a very small proportion of individuals with IPF undergo PSG highlights that this potentially helpful testing is being infrequently utilized.

Obviously, PSG in of itself is not responsible for the observed positive health outcomes. Rather, subsequent downstream events following PSG (i.e., diagnosis and treatment of sleep breathing disorders) are presumed to explain the observed benefits, with PSG simply reflecting a surrogate marker for these events. Although our study is the first population-based study demonstrating positive outcomes in IPF following PSG, our findings are consistent with the results of two earlier, smaller observational studies that showed improved survival among individuals with combined IPF and OSA in association with PAP adherence [15, 17]. Our results are also consistent with three, small observational studies reporting improvements in scores on multiple quality-of-life instruments in association with treatment of OSA in IPF [15, 17, 39]. Our findings of improved health outcomes differ from another observational study, where neither having OSA, nor being adherent with PAP therapy, generally influenced all-cause mortality or progression-free survival, although in this study only a minority of individuals with interstitial lung disease had IPF (32.5%) [26]. Although significant reductions in morbidity and mortality were observed in association with PSG receipt among individuals with IPF, only a very small proportion of our cohort (4.0%) underwent this beneficial testing, underscoring its wide under-utilization in the IPF population. Early identification and treatment of sleep breathing disorder may be important, as there is some evidence to suggest that more advanced degrees of sleep breathing disorder in IPF are associated with worse health outcomes [16].

To account for measured differences between our exposed and control groups, we performed rigorous propensity score matching, adequately balancing on 41 covariates, including on multiple markers on IPF severity, general health status, comorbidities and health care system utilization. Furthermore, the fact that, as with the exposed group, control group entry was based on receipt of a test (i.e., spirometry) serves to makes it less likely that changes in overall health status or health-seeking behaviour explain our results. Reduced respiratory-related hospitalization in association with PSG in the subgroups of individuals with prior history of respiratory-related hospitalization and previous systemic corticosteroid receipt (which are sicker subgroups of individuals) decreases the likelihood that our findings are as result of 'healthy user' bias. Small sample size may account for the fact that rate of all-cause was not significantly lower in association with PSG in the aforementioned two subgroups, and that significantly improved outcomes were not observed in the sensitivity analyses by CHF comorbidity, as point estimates were below 1.00 for all outcomes across all subgroups.

Our study has several limitations. Our study is hypothesis-generating and causation cannot be concluded as the explanation for our findings. Unmeasured differences between our exposed and control groups could explain our findings. Information on symptoms, lung function and exercise capacity measures, oxygenation status, and extent of fibrosis on imaging, were not available in our health administrative databases. While we balanced the exposed and control groups on a number of important indicators of IPF severity (including history of respiratory-related hospitalization, history of ICU admission, CHF, and previous systemic corticosteroid and respiratory antibiotic receipt), we acknowledge that these are not all validated severity markers. Any unmeasured IPF severity markers would most likely track disease severity markers that we did have information on, and these were in fact consistently more prevalent among exposed versus control individuals, before propensity score matching. Specifically, before propensity score matching, compared to controls, individuals undergoing PSG were more frequently admitted to hospital or ICU for respiratory-related reasons in the preceding year, had CHF and other forms of cardiovascular disease, had other pulmonary comorbidities, and received a systemic corticosteroid and a respiratory antibiotic. Any unmeasured IPF severity markers would logically be anticipated to track these, and if persistently unbalanced after propensity score matching, then bias the analysis against the exposed group, and yet, better health outcomes were observed in association with PSG receipt among these individuals. Our IPF identification algorithm, while developed by internationally-recognized IPF experts [1] and previously applied [1, 26–29], has not been validated, and we also lacked a validated measure of disease duration. The IPF algorithm largely relies on a J84.1 coding occurring in the context of a hospitalization or emergency room (ER) visit, which has the potential to under-capture individuals with milder degrees of disease, who would less likely present to hospital. Therefore, our results may not be applicable to all individuals with IPF. However, if individuals with milder degrees of IPF were less likely included in our study, this would only serve to decrease possible 'healthy user' bias influencing our results. Although we used PSG receipt as a marker for diagnosis and treatment of sleep breathing disorder, we acknowledge the limitations of this approach, that undergoing a PSG does not mean that sleep breathing disorder was indeed established in an individual or that any diagnosed sleep breathing disorder was being appropriately treated. However, given the high frequency with which sleep breathing disorders is known to occur among individuals with IPF [10–17], it logically follows that the vast majority of individuals in our control group will have undiagnosed/untreated sleep breathing disorder, and therefore, PSG receipt becomes not an unreasonable surrogate marker. A sensitivity analysis where receipt specifically of therapeutic PSG was used to identify exposed individuals (which may be a superior marker for diagnosis and treatment of sleep breathing disorder) could not be undertaken because of small sample size (only 12/201 [6%] of exposed individuals received a therapeutic PSG). The very low number of individuals undergoing therapeutic PSG is likely largely explained by the advent of auto-titrating PAP units, with data recording and download capabilities, which has substantially shifted airway pressure determination from lab to home. A sensitivity analysis by PAP or supplemental oxygen receipt was also not feasible because of small sample size (only 37/201 [18%] of our exposed individuals were recorded as having subsequently received PAP or supplemental oxygen) and this is as a result of known incomplete recording of these therapies in our health administrative databases. While we propose that our findings of better health outcomes in association with PSG testing are likely as a result of diagnosis and treatment of sleep breathing disorder, the institution of other cardio-pulmonary interventions/treatments as a consequence of PSG results may have also possibly contributed to observed improvements. It is possible that undergoing PSG testing is reflective of having a more thorough health care provider, and that the observed improved health outcomes in association with PSG are then as a consequence of receipt of more thorough overall medical care, rather than direct downstream consequences following PSG. However, our propensity score model included multiple markers of health care utilization, including number of outpatient physician visits in the preceding year, receipt of other types of investigations (including chest computed tomography, echocardiography and exercise oximetry testing) and receipt of multiple types of pharmacotherapies (including anti-fibrotic therapy, systemic corticosteroids, respiratory antibiotics, inhalers, smoking cessation drugs and cardiac medications), and exposed and controls were well-balanced on all these variables, thereby making it less likely that differences in overall medical care received explain our findings. Our health administrative databases also do not contain objective information relating to sleep breathing disorder diagnosis (e.g., apnea–hypopnea index, oxygen desaturation measures). Our findings also potentially do not apply to individuals with IPF under the age of 66 years old, who were excluded from our study.

Receipt of PSG was found to be associated with significantly lower rates of respiratory-related hospitalization and all-cause mortality among individuals with IPF. Furthermore, we observed that PSG testing was being infrequently performed in the IPF population. Our findings have potentially important implications for the management of IPF, a disease that is progressive and for which there are limited treatment options. While our study was limited by the use of PSG as a surrogate marker, our results raise the possibility that evaluation for and treatment of sleep breathing disorders is beneficial in IPF and that is management strategy is being suboptimally applied. Further research, particularly clinical trials, would be needed to exclude possible unresolved confounding and establish causation.

## Supplementary Information

**Additional file 1**. Online supplement.

## Data Availability

The datasets used in this study are held securely in coded form at ICES. Data sharing agreements prohibit ICES from making the datasets publicly available. However, dataset access may be granted to those who meet pre-specified criteria for confidential access by submitting a request either by phone or email to ICES (website: www.ices.on.ca). The full dataset creation plan and underlying analytic code can be made available by contacting the corresponding author (Dr. Nicholas Vozoris) by email, with the understanding that the computer programs may rely upon coding templates or macros that are unique to ICES and are therefore either inaccessible or may require modification.

## Abbreviations

CHF: Congestive heart failure
CIHI-DAD: Canadian Institute for Health Information Discharge Abstract Database
CI: Confidence interval
ER: Emergency room
HR: Hazard ratio
ICD-10: International Classification of Diseases Version 10
ICES: Institute for Clinical Evaluative Sciences
ICU: Intensive care unit
IPF: Idiopathic pulmonary fibrosis
NACRS: National Ambulatory Care Reporting System
NNT: Number needed to treat
ODB: Ontario Drug Benefit
OHIP: Ontario Health Insurance Plan
OSA: Obstructive sleep apnea
PAP: Positive airway pressure
PSG: Polysomnography
