# Supplementary material for: Morbidity and mortality reduction associated with polysomnography testing in idiopathic pulmonary fibrosis: a population-based cohort study
Source: BMC Pulm Med. 2021 Jun 2;21:185. doi: 10.1186/s12890-021-01555-x (PMC8170825; doi:10.1186/s12890-021-01555-x)
Supplement: Supplementary file 1 — Additional file 1. Online supplement. [file 12890_2021_1555_MOESM1_ESM.doc]

**ONLINE SUPPLEMENT**

**for**

**MORBIDITY AND MORTALITY REDUCTION ASSOCIATED WITH POLYSOMNOGRAPHY TESTING IN IDIOPATHIC PULMONARY FIBROSIS: A POPULATION-BASED COHORT STUDY**

**Nicholas T Vozoris^a,b,c,d^, Andrew Wilton^d^, Peter C Austin^d,e^, Tetyana Kendzerska**^d,f^**,**

**Clodagh M Ryan^c,g^, Andrea S Gershon^c,d,e,h^**

^a^ Division of Respirology, Department of Medicine, St. Michael’s Hospital, Toronto, Ontario, Canada.

^b^ Keenan Research Centre in the Li Ka Shing Knowledge Institute, St Michael’s Hospital, Toronto, Ontario, Canada

^c^ Department of Medicine, University of Toronto, Toronto, Ontario, Canada.

^d^ ICES, Toronto, Ontario, Canada.

^e^ Institute of Health Policy, Management, and Evaluation, University of Toronto, Toronto, Ontario, Canada

^f^The Ottawa Hospital Research Institute, University of Ottawa, Ottawa, Ontario, Canada

^g^Division of Respirology, University Health Network, Toronto, Ontario, Canada

^h^Sunnybrook Hospital, Department of Medicine, Toronto, Ontario, Canada

**Address for correspondence:** Dr. Nicholas Vozoris, Division of Respirology, Department of Medicine, St. Michael’s Hospital, 30 Bond Street, Toronto, Ontario, Canada, M5B 1W8. Phone: 416-864-6026; Fax: 416-864-5649; Email: nick.vozoris@utoronto.ca

**CONTENTS**

1. List and description of additional ICES databases used in this study (page 3)

2. OHIP codes designating computed tomography chest imaging, lung biopsy and bronchoscopy (page 4)

3. List of ICD-10 codes for other forms of interstitial lung disease (page 5)

4. OHIP codes designating laboratory-based polysomnography and spirometry testing (page 7)

5. Identification of lung transplantation (page 8)

6. Cohort baseline characteristics, before and after propensity score matching (full list of variables included) (page 9)

7. Additional sensitivity analysis (page 11)

**Section 1: List and description of additional ICES databases used in this study**

1. Assistive Devices Program (ADP) database: contains information on the provision of some types of assistive medical devices, including continuous positive airway pressure (CPAP) therapy and supplemental oxygen.

2. Chronic obstructive pulmonary disease (COPD) database: contains individuals with validated physician-diagnosed COPD [1]

2. Congestive heart failure (CHF) database: contains individuals with validated physician-diagnosed CHF [2]

3. Hypertension database - contains individuals with validated physician-diagnosed hypertension [3]

3. Ontario Cancer Registry (OCR): a validated provincial cancer registry [4]

4. Ontario Diabetes Dataset (ODD): contains individuals with validated physician-diagnosed diabetes [5]

5. Ontario Mental Health Reporting System (OMHRS): contains information on all mental health hospital admissions

6. Ontario Myocardial Infarction Dataset (OMID): contains individuals with acute myocardial infarctions that were associated with hospitalization [6]

**REFERENCES:**

1. Gershon AS, Wang C, Guan J, Vasilevska-Ristovska J, Cicutto L, To T. Identifying individuals with physician diagnosed COPD in health administrative databases. J COPD. 2009;6:388-94.

2. [Schultz SE](http://www.ncbi.nlm.nih.gov/pubmed/?term=Schultz SE%5BAuthor%5D&cauthor=true&cauthor_uid=23735455), [Rothwell DM](http://www.ncbi.nlm.nih.gov/pubmed/?term=Rothwell DM%5BAuthor%5D&cauthor=true&cauthor_uid=23735455), [Chen Z](http://www.ncbi.nlm.nih.gov/pubmed/?term=Chen Z%5BAuthor%5D&cauthor=true&cauthor_uid=23735455), Tu K. Identifying cases of congestive heart failure from administrative data: a validation study using primary care patient records. [Chronic Dis Inj Can](http://www.ncbi.nlm.nih.gov/pubmed/23735455). 2013;33:160-6.

3. Tu K, Campbell NRC, Chen Z, [Cauch-Dudek KJ](https://www.ncbi.nlm.nih.gov/pubmed/?term=Cauch-Dudek KJ%5BAuthor%5D&cauthor=true&cauthor_uid=20101286), [McAlister FA](https://www.ncbi.nlm.nih.gov/pubmed/?term=McAlister FA%5BAuthor%5D&cauthor=true&cauthor_uid=20101286). Accuracy of administrative databases in identifying patients with hypertension. Open Medicine. 2007; 1: e5-7.

4. [Robles SC](http://www.ncbi.nlm.nih.gov/pubmed/?term=Robles SC%5BAuthor%5D&cauthor=true&cauthor_uid=3367181), [Marrett LD](http://www.ncbi.nlm.nih.gov/pubmed/?term=Marrett LD%5BAuthor%5D&cauthor=true&cauthor_uid=3367181), [Clarke EA](http://www.ncbi.nlm.nih.gov/pubmed/?term=Clarke EA%5BAuthor%5D&cauthor=true&cauthor_uid=3367181), [Risch HA](https://www.ncbi.nlm.nih.gov/pubmed/?term=Risch HA%5BAuthor%5D&cauthor=true&cauthor_uid=3367181). An application of capture-recapture methods to the estimation of completeness of cancer registration. J Clin Epidemiol. 1988;41:495-501.

5. Hux JE, Ivis F, Flintoft V, Bica A. Diabetes in Ontario: determination of prevalence and incidence using a validated administrative data algorithm. Diabetes Care. 2002;25:512-6.

6. Austin PC, Daly PA, Tu JV. A multicenter study of the coding accuracy of hospital discharge administrative data for patients admitted to cardiac care units in Ontario. Am Heart J 2002;144:290-6.

**Section 2: OHIP codes designating computed tomography chest imaging, lung biopsy and bronchoscopy**

| **Investigation** | **OHIP codes** |
| --- | --- |
| Computed tomography chest scan | X406, X407, X125, X235 |
| Lung biopsy (including transbronchial biopsy, surgical lung biopsy, or endobronchial ultrasound and biopsy) | E638, Z333, Z338, M137, G050 |
| Bronchoscopy | Z327, Z330 |

**Section 3: List of ICD-10 codes for other forms of interstitial lung disease***

| **ICD-10 code** | **Description** |
| --- | --- |
| D76 | | Other specified diseases with participation of lymphoreticular and reticulohistiocytic tissue | | --- | |
| D86 | Sarcoidosis |
| E75.2, .5, .6 | | Other sphingolipidosis, Other lipid storage disorders, Lipid storage disorder not specified | | --- | |
| E83.1 | | Disorders of iron metabolism | | --- | |
| E85 | Amyloidosis |
| E88.8 | | Other specified metabolic disorders (Launois-Bensaude adenolipomatosis, Trimethylaminuria) | | --- | |
| H42.0 | | Glaucoma in endocrine, nutritional and metabolic diseases (e.g., amyloidosis) | | --- | |
| I68.0 | | Cerebral amyloid angiopathy | | --- | |
| J60 | | Coalworker pneumoconiosis | | --- | |
| J61 | | Pneumoconiosis due to asbestos and other mineral fibres | | --- | |
| J62 | | Pneumoconiosis due to dust containing silica | | --- | |
| J64 | | Unspecified pneumoconiosis | | --- | |
| J65 | | Pneumoconiosis associated with tuberculosis | | --- | |
| J66 | | Pneumoconiosis due to other inorganic dusts | | --- | |
| J67 | | Hypersensitivity pneumonitis due to organic dust | | --- | |
| J68.4 | | Chronic respiratory conditions due to chemicals, gases, fumes and vapours | | --- | |
| J68.8 | | Other respiratory conditions due to chemicals, gases, fumes and vapours | | --- | |
| J70 | | Respiratory conditions due to other external agents | | --- | |
| J82 | | Pulmonary eosinophilia, not elsewhere classified | | --- | |
| J84.0 | | Alveolar and parietoalveolar conditions | | --- | |
| J84.8 | | Other specified interstitial pulmonary diseases | | --- | |
| J84.9 | | Interstitial pulmonary disease, unspecified | | --- | |
| J99.1 | | Respiratory disorders in other diffuse connective tissue disorders | | --- | |
| J99.8 | | Respiratory disorders in other diseases classified elsewhere | | --- | |
| K50 | | Crohn's disease of small intestine | | --- | |
| M05 | | Seropositive rheumatoid arthritis | | --- | |
| M06 | | Other rheumatoid arthritis | | --- | |
| M08 | | Juvenile arthritis | | --- | |
| M12 | | Chronic postrheumatic arthropathy (Jaccoud) | | --- | |
| M30.1 | | Polyarteritis with lung involvement (Churg-Strauss) | | --- | |
| M31.0 | | Hypersensitivity angiitis (Goodpasture’s syndrome) | | --- | |
| M31.3 | Wegener granulomatosis |
| M32 | | Systemic lupus erythematosus | | --- | |
| M33 | | Dermatopolymyositis | | --- | |
| M34 | | Systemic sclerosis | | --- | |
| M35 | | Other systemic involvement of connective tissue | | --- | |
| M46 | | Other inflammatory spondylopathies | | --- | |
| Q85.1 | | Tuberous sclerosis | | --- | |

*Taken from: [Hopkins RB](https://www.ncbi.nlm.nih.gov/pubmed/?term=Hopkins RB%5BAuthor%5D&cauthor=true&cauthor_uid=27230442), [Burke N](https://www.ncbi.nlm.nih.gov/pubmed/?term=Burke N%5BAuthor%5D&cauthor=true&cauthor_uid=27230442), [Fell C](https://www.ncbi.nlm.nih.gov/pubmed/?term=Fell C%5BAuthor%5D&cauthor=true&cauthor_uid=27230442), [Dion](https://pubmed.ncbi.nlm.nih.gov/?sort=date&size=200&term=Dion+G&cauthor_id=27230442) G, [Kolb](https://pubmed.ncbi.nlm.nih.gov/?sort=date&size=200&term=Kolb+M&cauthor_id=27230442) M. Epidemiology and survival of idiopathic pulmonary fibrosis from national data in Canada. [Eur Respir J](https://www.ncbi.nlm.nih.gov/pubmed/27230442). 2016;48(1):187-95.

**Section 4: OHIP codes designating laboratory-based polysomnography (PSG) and spirometry testing**

**Laboratory-based PSG:**

Any of the following OHIP codes were used to designate laboratory-based PSG testing receipt, and therefore, exposed group entry:

1. J896, J696, J897, J697, J890 or J690: these codes designate diagnostic sleep studies

2. J895, J695, J889 or J689: these codes designate therapeutic sleep studies

3. J898, J899 or J890: these codes designate incomplete overnight sleep studies (where less than four hours of sleep was attained by the patient). Incomplete sleep studies were considered, because even though a limited amount of sleep would have been attained, in some instances, there may have still been enough sleep to establish a sleep breathing disorder diagnosis and then initiation of appropriate treatment.

**Spirometry:**

Any of the following OHIP codes were used to designate receipt of spirometry:

1. J301: codes for simple spirometry, volume versus time study

2. J304: codes for simple spirometry, volume versus time study, after bronchodilator challenge

3. J324: codes for simple spirometry, volume versus flow study

4. J327: codes for simple spirometry, volume versus flow study, after bronchodilator challenge

**Section 5: Identification of lung transplantation**

Lung transplantation was defined by a hospitalization, associated with one of the following intervention codes: 1GR85 (transplant of lung lobes), 1GT85 (lung transplant) or 1HY85 (heart and lung transplantation). The intervention code also had to be coded as ‘not abandoned’.

**Section 6: Cohort baseline characteristics, before and after propensity score matching (full list of variables included*)**

| **Baseline characteristics** | **Prior to propensity score matching** | | | **After propensity score matching** | | |
| --- | --- | --- | --- | --- | --- | --- |
|  | **Exposed**  **N=201** | **Controls**  **N=4843** | **Standardized difference**† | **Exposed**  **N=189** | **Controls**  **N=189** | **Standardized difference**† |
| **Age (mean + SD)** | 75.9 ± 6.2 | 78.3 ± 6.8 | 0.37 | 76.2 ± 6.1 | 76.4 ± 6.8 | 0.04 |
| **Women (%)** | 33.8 | 45.1 | 0.23 | 33.3 | 33.3 | 0.00 |
| **Low income as per ODB (%)** | 12.9 | 18.1 | 0.14 | 13.2 | 13.8 | 0.02 |
| **Rural residence (%)** | 17.4 | 14.2 | 0.09 | 15.9 | 20.1 | 0.11 |
| **Long-term care residence (%)** | ‡ | 1.9 | 0.01 | ‡ | ‡ | 0.04 |
| **Respiratory-related hospitalization past year (%)** | 19.9 | 9.1 | 0.31 | 18.5 | 18.5 | 0.00 |
| **ICU admission during respiratory-related hospitalization past year (%)** | 4.0 | 1.5 | 0.15 | 4.2 | 4.8 | 0.03 |
| **Congestive heart failure** | 46.3 | 29.8 | 0.34 | 45.5 | 45.5 | 0.00 |
| **Systemic corticosteroid receipt past year (%)** | 37.3 | 33.0 | 0.09 | 37.0 | 37.0 | 0.00 |
| **Respiratory antibiotic receipt past year (%)** | 72.1 | 66.5 | 0.12 | 72.0 | 73.5 | 0.04 |
| **Anti-fibrotic drug**§ **receipt past year (%)** | ‡ | 0.9 | 0.09 | ‡ | ‡ | 0.00 |
| **Total number outpatient visits past year (mean + SD)** | 19.2 ± 10.2 | 15.7 ± 9.2 | 0.36 | 18.9 ± 10.0 | 18.1 ± 9.3 | 0.09 |
| **Total number hospitalizations past year (mean + SD)** | 0.9 ± 1.3 | 0.6 ± 1.0 | 0.32 | 0.9 ± 1.3 | 1.0 ± 1.5 | 0.02 |
| **Total number ICU admissions past year (mean + SD)** | 0.1 ± 0.4 | 0.1 ± 0.3 | 0.09 | 0.1 ± 0.4 | 0.1 ± 0.4 | 0.01 |
| **Any surgery past year (%)** | 11.4 | 8.7 | 0.09 | 10.6 | 11.6 | 0.03 |
| **CT Chest scan past year (%)** | 67.7 | 54.7 | 0.27 | 66.7 | 69.3 | 0.06 |
| **Echocardiogram past year (%)** | 65.7 | 43.8 | 0.45 | 64.0 | 64.6 | 0.01 |
| **Exercise oximetry past year (%)** | 38.3 | 31.8 | 0.14 | 37.6 | 37.6 | 0.00 |
| **Pulmonary embolism**ǁ **(%)** | 6.0 | 3.2 | 0.13 | 6.3 | 6.9 | 0.02 |
| **COPD (%)** | 70.6 | 67.6 | 0.07 | 69.8 | 68.8 | 0.02 |
| **Other pulmonary disease**ǁ^¶^ **(%)** | 94.5 | 87.4 | 0.25 | 94.2 | 94.7 | 0.02 |
| **GERD**ǁ **(%)** | 6.5 | 5.2 | 0.06 | 6.3 | 5.8 | 0.02 |
| **Myocardial infarction (%)** | 14.9 | 10.0 | 0.15 | 14.3 | 16.4 | 0.06 |
| **Hypertension (%)** | 84.1 | 77.5 | 0.17 | 83.6 | 82.5 | 0.03 |
| **Atherosclerosis**ǁ **(%)** | 4.0 | 3.6 | 0.02 | 3.7 | 5.8 | 0.10 |
| **Diabetes (%)** | 41.3 | 32.4 | 0.19 | 41.3 | 35.4 | 0.12 |
| **Stroke or TIA**ǁ **(%)** | 6.5 | 8.1 | 0.06 | 6.9 | 9.0 | 0.08 |
| **Any cancer (%)** | 19.9 | 21.3 | 0.03 | 19.6 | 18.5 | 0.03 |
| **Liver disease**ǁ **(%)** | 4.0 | 3.9 | 0.01 | 3.7 | 4.8 | 0.05 |
| **Kidney disease**ǁ **(%)** | 40.8 | 27.5 | 0.28 | 40.2 | 45.5 | 0.11 |
| **Psychotic mental health disease**ǁ **(%)** | 7.5 | 4.2 | 0.14 | 7.9 | 7.9 | 0.00 |
| **Non-psychotic mental health disease**ǁ **(%)** | 74.6 | 36.2 | 0.84 | 73.0 | 70.9 | 0.05 |
| **Smoking cessation medication** receipt past year(%)** | ‡ | 1.4 | 0.05 | ‡ | ‡ | 0.08 |
| **Any inhaler receipt past year (%)** | 49.3 | 43.2 | 0.12 | 50.3 | 51.9 | 0.03 |
| **Benzodiazepine receipt past 3 months (%)** | 16.4 | 17.0 | 0.02 | 16.9 | 16.4 | 0.01 |
| **Opioid receipt past 3 months (%)** | 17.9 | 17.8 | 0.00 | 19.0 | 19.0 | 0.00 |
| **Diuretic medication receipt past 3 months (%)** | 36.8 | 23.8 | 0.29 | 34.4 | 37.0 | 0.06 |
| **Nitrate medication receipt past 3 months (%)** | 11.4 | 8.7 | 0.09 | 11.6 | 13.2 | 0.05 |
| **Antiplatelet or anticoagulant medication receipt past 3 months (%)** | 33.3 | 24.8 | 0.19 | 33.3 | 39.7 | 0.13 |
| **Other cardiac drug**††**receipt past 3 months (%)** | 77.6 | 67.3 | 0.23 | 77.2 | 77.8 | 0.01 |
| **Year of cohort entry (%)** |  |  |  |  |  |  |
| 2007-2008 | 7.0 | 11.9 | 0.17 | 6.9 | 7.9 | 0.04 |
| 2009 | 9.0 | 11.3 | 0.08 | 9.0 | 7.9 | 0.04 |
| 2010 | 4.5 | 11.5 | 0.26 | 4.8 | 5.8 | 0.05 |
| 2011 | 8.0 | 11.1 | 0.11 | 8.5 | 10.1 | 0.05 |
| 2012 | 9.0 | 9.6 | 0.02 | 9.5 | 12.2 | 0.09 |
| 2013 | 10.4 | 10.6 | 0.01 | 11.1 | 6.3 | 0.17 |
| 2014 | 10.9 | 9.0 | 0.06 | 11.1 | 8.5 | 0.09 |
| 2015 | 14.9 | 9.0 | 0.18 | 15.3 | 15.9 | 0.01 |
| 2016 | 10.0 | 8.7 | 0.04 | 10.6 | 8.5 | 0.07 |
| 2017 | 16.4 | 7.3 | 0.28 | 13.2 | 16.9 | 0.10 |

COPD = chronic obstructive pulmonary disease; GERD = gastroesophageal reflux; ICU = intensive care unit; ODB = Ontario Drug Benefit; SD = standard deviation; TIA = transient ischemic attack

*An abridged version of this table is found in the manuscript proper

†Standardized differences of > 0.10 are thought to indicate potentially meaningful differences

‡Data has been suppressed, according to ICES guidelines, because of small sample size

§Includes Pirfenidone and Nintedanib

ǁPresence of comorbidities was based on 3-year look-back from the index date

^¶^Includes asthma, bronchiectasis, occupational lung disease, pleural effusion, interstitial disease, pneumothorax, atelectasis and other

******Includes Wellbutrin and Varenicline

††Includes beta-blockers, calcium channel blockers, angiotensin converting enzyme inhibitors (ACEI), and angiotension receptor blockers (ARB)

**Section 7: Additional sensitivity analysis**

A final sensitivity analysis that was undertaken was evaluating our outcomes stratifying by sex, to assess if possible health benefits of undergoing PSG extended to women with IPF, given that IPF is known to occur with increased frequency among men. Among men, there was no significant difference in rate of respiratory-related hospitalization in association with PSG receipt, but there was significantly reduced rate of all-cause mortality (HR 0.41, 95% CI 0.23-0.73, p=0.003) among those that underwent PSG relative to controls (see table below). Among women, no significant differences in respiratory-related hospitalization or all-cause mortality were observed in association with PSG receipt. However, it is worthy to note that the point estimates for respiratory-related hospitalization and all-cause mortality among women undergoing PSG were <1.00 and that there were smaller sample size numbers among women than men.

**Hazard ratios (HR) and confidence intervals (CI) for outcomes in the propensity-score matched cohort, stratifying by sex**

| **Sex** | **Outcomes** | **Exposure status** | **Number of events (%)** | **HR (95% CI)** | **p-value** |
| --- | --- | --- | --- | --- | --- |
|  |  |  |  |  |  |
| Women | Respiratory-related hospitalization | Exposed | * | 0.35  (0.10-1.24) | 0.11 |
|  |  | Controls | 8 (12.3) | 1.00 |  |
|  |  |  |  |  |  |
|  | All-cause mortality | Exposed | 7 (10.8) | 0.60  (0.22-1.62) | 0.31 |
|  |  | Controls | 11 (16.9) | 1.00 |  |
|  | | | | | |
| Men | Respiratory-related hospitalization | Exposed | 10 (8.1) | 0.53  (0.27-1.05) | 0.07 |
|  |  | Controls | 20 (16.3) | 1.00 |  |
|  |  |  |  |  |  |
|  | All-cause mortality | Exposed | 13 (10.6) | 0.41  (0.23-0.73) | 0.003 |
|  |  | Controls | 31 (25.2) | 1.00 |  |

* Data has been suppressed, according to ICES guidelines, because of small sample size
